# Differential expression of Vitamin D binding protein in thyroid cancer health disparities

**DOI:** 10.18632/oncotarget.27920

**Published:** 2021-03-30

**Authors:** Brittany Mull, Ryan Davis, Iqbal Munir, Mia C. Perez, Alfred A. Simental, Salma Khan

**Affiliations:** ^1^Harbor UCLA Medical Center, Torrance, CA 90502, USA; ^2^Division of Biochemistry, Loma Linda, CA 92350, USA; ^3^Center for Health Disparities & Molecular Medicine, Loma Linda, CA 92350, USA; ^4^Riverside University Health System, Moreno Valley, CA 92555, USA; ^5^Department of Pathology & Human Anatomy, Loma Linda University School of Medicine, Loma Linda, CA 92354, USA; ^6^Department of Otolaryngology, Loma Linda University School of Medicine, Loma Linda, CA 92354, USA; ^7^Department of Internal Medicine, Loma Linda University School of Medicine, Loma Linda, CA 92354, USA

**Keywords:** DBP, thyroid cancer, health disparities

## Abstract

Thyroid cancer incidence, recurrence, and death rates are higher among Filipino Americans than European Americans. We propose that vitamin D binding protein (DBP) with multifunctionality with ethnic variability plays a key role within different ethnicities. In this study, we determined the correlation between differential DBP expression in tumor tissues and cancer staging in Filipino Americans versus European Americans. We assayed DBP expression by immunohistochemistry and analyzed the data with confocal microscopy on 200 thyroid cancer archival tissue samples obtained from both ethnicities. DBP-stable knockdown/gain-in-function assays were done by using DBP-shRNA/DBP-cDNA-expression *in vitro*. The majority of Filipino Americans presented with advanced tumor staging. In contrast, European Americans showed early staging and very few advanced tumors. A significantly low to no DBP staining was detected and correlated to the advanced staging in Filipino Americans. On the contrary, in the tumor tissues derived from European Americans, moderate to strong DBP staining was detected and correlated to early staging. When downregulation of the DBP gene in papillary thyroid cancer (PTC) cell lines was observed, tumor proliferation and migration were enhanced. On the other hand, the upregulation of the DBP gene decreased cell proliferation and migration in PTC cells. In conclusion, we determined a differential expression of an essential biological molecule (DBP) is linked to cancer staging in thyroid cancer health disparities in two ethnicities. Loss-of-DBP/gain-in-DBP-function influenced tumor progression. A future study is underway to determine the DBP regulation and its downstream pathways to elucidate strategies to eliminate the observed thyroid cancer health disparities.

## INTRODUCTION

Thyroid cancer is one of the most prevalent endocrine cancers [1–4]. An epidemic of thyroid cancer (TC) in California was reported by the California-based Cancer Prevention Institute [2, 5–9]. According to the California Cancer Registry, TC incidence is higher in Filipino Americans than European Americans or other Asian Americans [10–16]. Although it is believed that there is an actual increase in thyroid cancer incidence due to changes in risk factors [17–21], the exact mechanism of this steady increase remains unknown. According to the analysis of ethnicity and geographical residence, variations in thyroid cancer incidence may be attributed to local environmental influences and genetic/biological alterations [10, 22, 23]. However, currently no mechanism explains the observed increase in incidence, recurrence, and death rate among Filipino Americans with thyroid. We identified a highly polymorphic protein, called vitamin D binding protein (DBP) that could play an important role in thyroid cancer progression in ethnically predisposed group. Because of its highly polymorphic nature in humans [24–28], a structural/functional defect of DBP gene could contribute to thyroid cancer development and malignant transformation.

A recent study demonstrated that vitamin D binds with DBP with high affinity under physiologic conditions to facilitate its bioavailability [29]. Although DBP has both vitamin D-dependent/independent roles in cancer development [30–35], vitamin D-dependent DBP functions in cancer are well studied with inconclusive results. Therefore, we tested whether DBP has the vitamin D-independent correlations/functions to thyroid cancer oncogenesis. Recent studies have shown that the human serum DBP has many physiologically important functions, ranging from transporting vitamin D metabolites, binding, and sequestering globular actin, binding fatty acids to possible roles in inflammation, and the immune response [29]. Although DBP is a polymorphic protein, functional implications are still mostly unknown. DBP showed several biologic mechanisms relevant to enhanced cancer risk [32, 35, 36]. DBP has anti-inflammatory and immunoregulatory functions and plays a role in several chronic diseases, including breast cancer. For example, when deglycosylated by T and B-cell glycosidases, DBP is involved in macrophage activation in the form of a DBP-macrophage activating factor (DBP-MAF) [37]. DBP is also involved in apoptosis and angiogenesis [38]. DBP level has been correlated with the prognosis of many cancers, including TC [30, 34, 35, 39–41]; the higher the DBP levels, the better the prognosis. Although DBP is an essential protein with multifunctional properties, [28, 41–47], very few studies are available on its contribution to thyroid cancer oncogenesis.

Since DBP gene variants showed differential expression across ethnicities [25, 40, 48, 49], DBP level in the tumor microenvironment may implicate the difference in TC prognosis between Filipino and European Americans. In the present study, we determined the differential expression of DBP protein in the thyroid cancer tissues and correlated it to cancer staging in Filipino Americans compared to European Americans. We also determined whether Knockdown/gain-in-DBP-function in thyroid cancer cell lines further enhanced/decreased cell proliferation and invasion capacities. This study concludes that the loss-of-DBP-function in the tumor tissues may stimulate an intracellular immune-modulating signaling pathway in thyroid cancer oncogenesis in Filipino Americans.

## RESULTS

### Differential expression of DBP protein in Filipino Americans vs. European Americans

**Figure 1:**
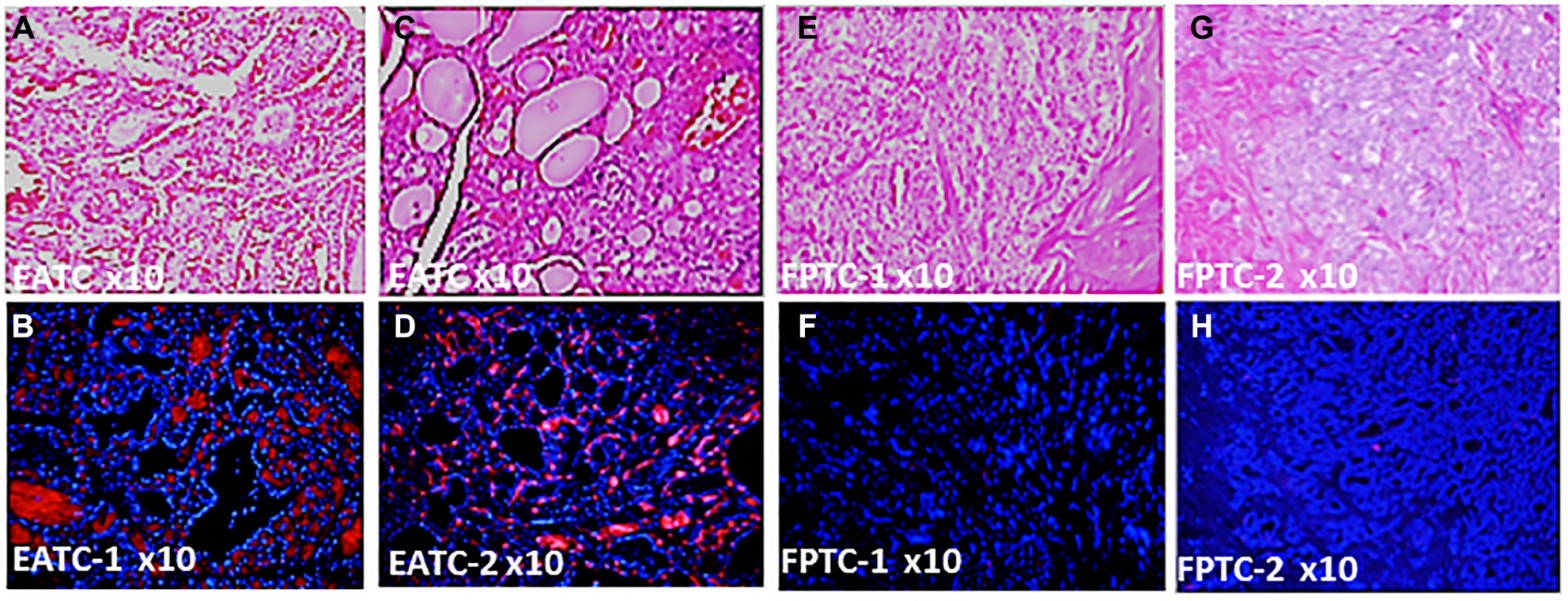
Differential expression of DBP protein in Filipino Americans vs. European Americans. (**A**) Hematoxylin & Eosin Staining of PTC from the representative tissue samples of EATC-1; (**B**) immunohistochemistry of DBP in EATC-1; (**C**) hematoxylin & Eosin Staining of PTC from EATC-2; (**D**) immunohistochemistry of DBP in EATC-2; (**E**) Hematoxylin & Eosin Staining of PTC from the representative tissue samples of FATC-1; (**F**) immunohistochemistry of DBP in FATC-1; (**G**) hematoxylin & Eosin Staining of PTC from FATC-2; (**H**) immunohistochemistry of DBP in FATC-2. Red, positive for DBP; blue, DAPI for nuclear stain. DBP, vitamin D binding protein; PTC, papillary thyroid cancer; EATC/FATC, European American/Filipino American thyroid Cancer. Magnification, 10×.

**Figure 2:**
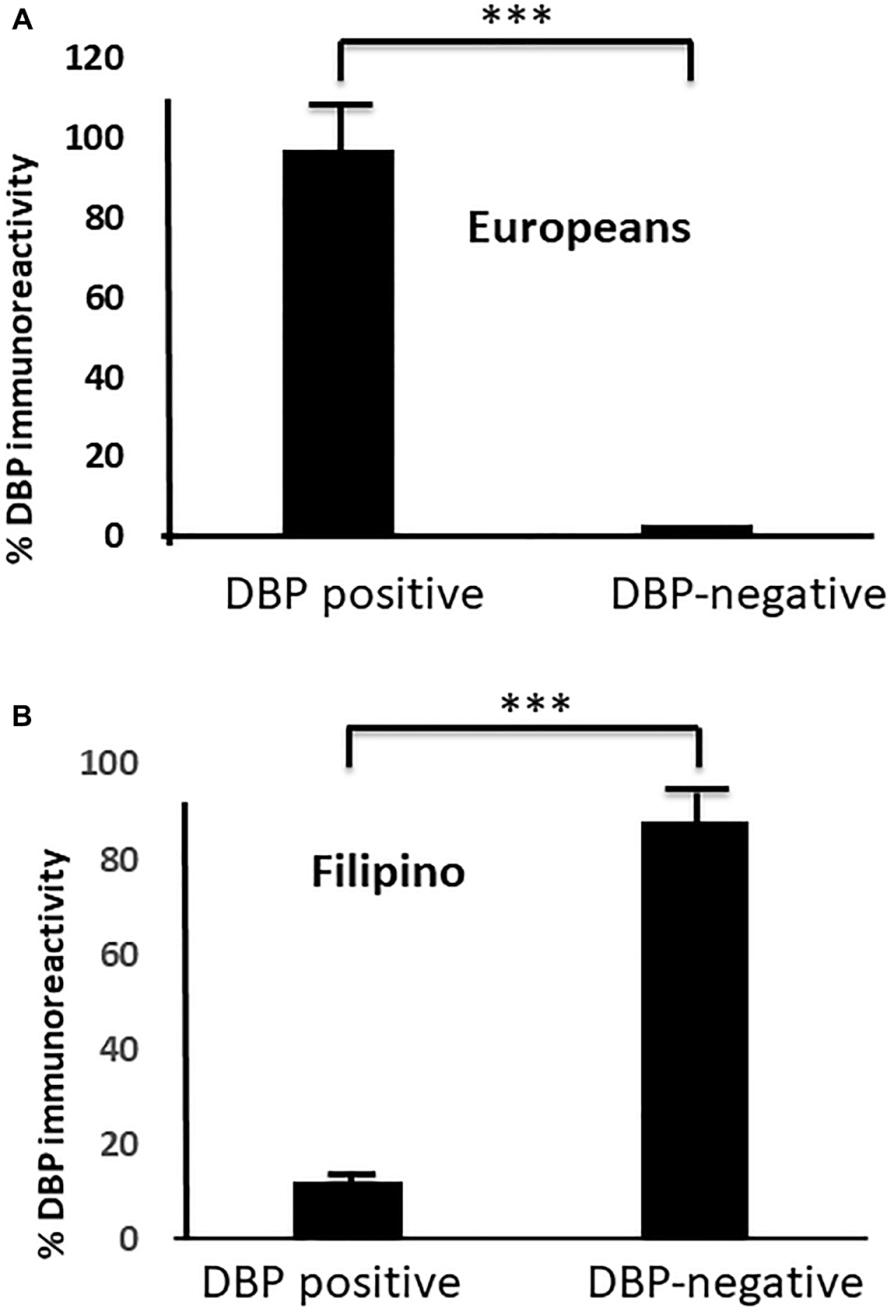
Differential DBP expression in FATC versus EATC. (**A**) a fewer number of DBP positive than negative samples in FATC; (**B**) a larger number of DBP positive samples DBP negative samples in EATC (^***^ *p* < 0.01, statistically significant) are shown. Moderate (++) to stronger (+++) DBP staining correlates to early staging (T1/T2) in EATC, whereas lower (+) to no DBP staining (0) correlates to advanced (T3/T4) staging in FATC. DBP, vitamin D binding protein; PTC, papillary thyroid cancer; EATC European American-derived thyroid cancer; FATC, Filipino American-derived thyroid cancer.

We selected papillary thyroid cancer (PTC) tissues to keep the genetic uniformity across the ethnicities. We confirmed histological diagnosis by H&E (Figure 1A, 1C, 1E and 1G), (Supplementary Figure 2A, 2C, 2E, 2G, 2I, 2K, 2M and 2O). Our demographic data showed disparities in sex, BMI, and pTNM staging of thyroid cancer (Supplementary Figure 1A–1D), (Supplementary Tables 1–3) with no age disparities (not shown) between Filipino Americans vs. European Americans. We evaluated the DBP staining intensity in thyroid cancer tissues derived from FA (FPTC) and EA (EATC). A weak (1+, *n* = 5) moderate (2++, *n* = 40) to strong (3+++, *n* = 55) DBP positivity was observed in most of were observed throughout the FATC (Figure 1B and 1D). In contrast, negative (0, *n* = 90) to weak (1+, *n* = 10) staining patterns were observed throughout the FATC (Figure 1F and 1H), Some of them showed very weak to total loss of DBP expression (Supplementary Figure 2B, 2D, 2F, 2H, 2J, 2L, 2N and 2P), and consulted the patient chart for demographic data and cancer staging. Out of 100, 55% of EATC showed (3+++), 40% (2++), and 5% (1+), whereas in FATC, 90% showed no (0), 10% with weak (1+) (Figure 2A–2B). All were statistically significant (^*^ *p* < 0.05).

### Correlation of DBP expression to thyroid cancer staging in Filipino Americans vs. European Americans

**Figure 3:**
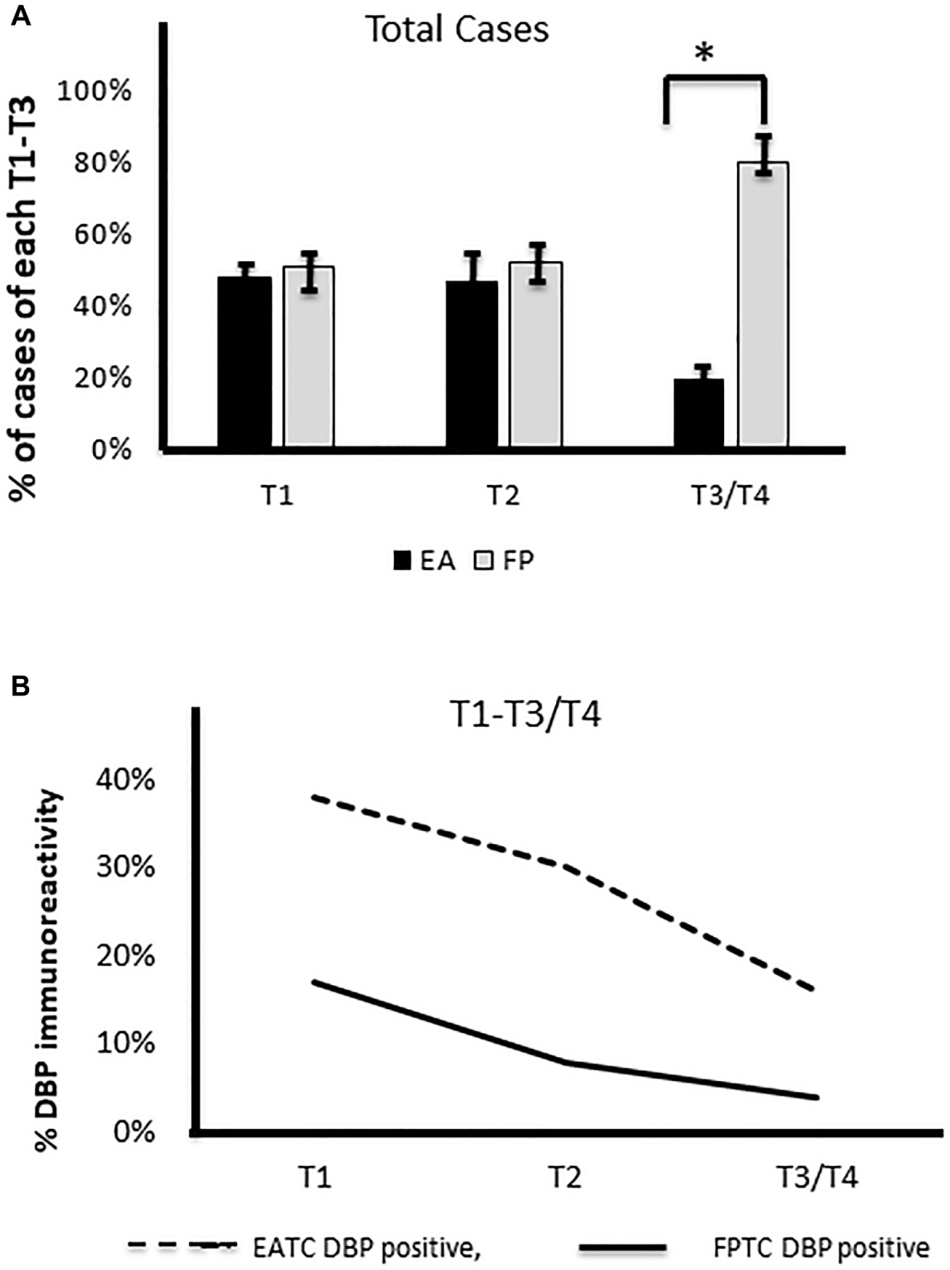
Correlation of DBP staining with staging. (**A**) A significantly higher number of cases with advanced staging (T3/T4) in Filipino Americans compared to European Americans (^*^ *p* < 0.01, statistically significant). (**B**) Lower the immunoreactivity of DBP (%), the higher the tumor staging in Filipino Americans. DBP, vitamin D binding protein.

In the beginning, we evaluated whether there was any correlation of sex in FA vs. EA. To do this, we first found females affected in both races. When we compared sex and BMI in both ethnicities (Supplementary Figure 1A and 1B), in both ethnic group, females are affected more frequently than males and we found a significantly higher BMI (85%) in FA patients compared to EA patients (28%). When we compared this to tumor size between FA vs. EA, we found a higher percentage of T3/T4 was noted in FA than EA patients. More node-positive tumors were shown in FA (N1a and N1b) in FA compared to EA patients (Supplementary Figure 1C and 1D). We also compared DBP staining intensities in FA to cancer staging, which we found inversely correlated with staging, i.e., the weaker or no DBP staining correlated to advanced staging, whereas in EA patient samples, moderate to strong staining was observed in early staging of PTC (Figure 3A and 3B). We found no correlation of their age/sex/BMI to DBP staining intensity. We compared the DBP staining pattern with TC staging; we found a significant inverse correlation to advance staging in FATC i.e., low (+) to no (0) DBP in advance staging), whereas moderate (++) to strong (+++) DBP accumulation was observed in early staging of thyroid cancer in EATC with stronger staining in tumor vessels, stromal cells, and thyroid cancer tissues. We found no correlation of DBP staining with age, sex, or BMI in both ethnicities.

### The effects of loss-of-/gain-in-function of DBP gene in papillary thyroid cancer cells

**Figure 4:**
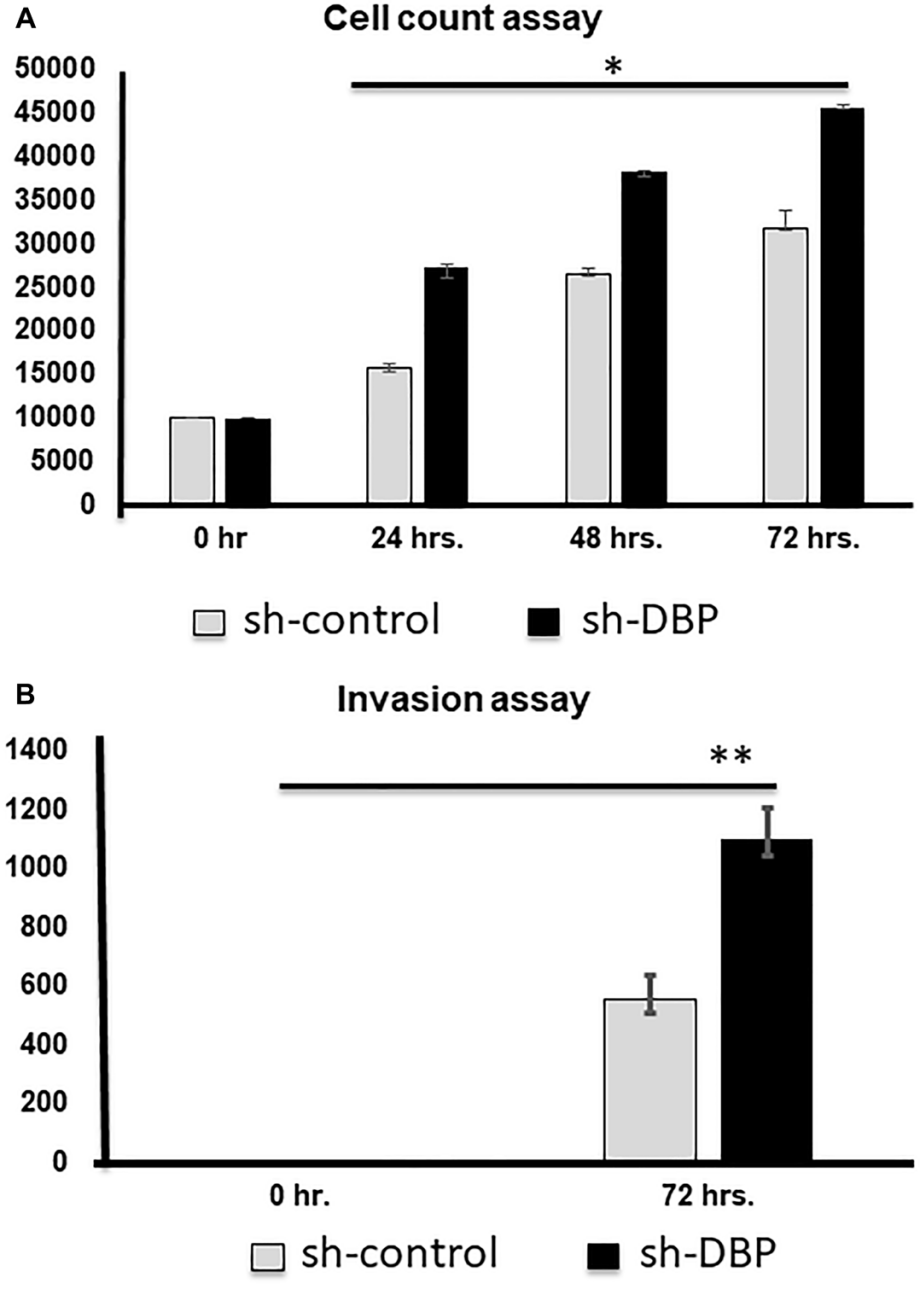
Cell counting and invasion assays after si-DBP-knockdown. (**A**) Cell counting at 0, 24, 48, and 72 hrs. Knock-down, a significantly (^*^ *p* < 0.05) higher cell rescue occurred with si-DBP-knockdown compared to si-scramble control. (**B**) A significantly higher invasion occurred after si-DBP-knock-down compared to si-control at 72 hrs. (^**^ *p* < 0.01).

**Figure 5:**
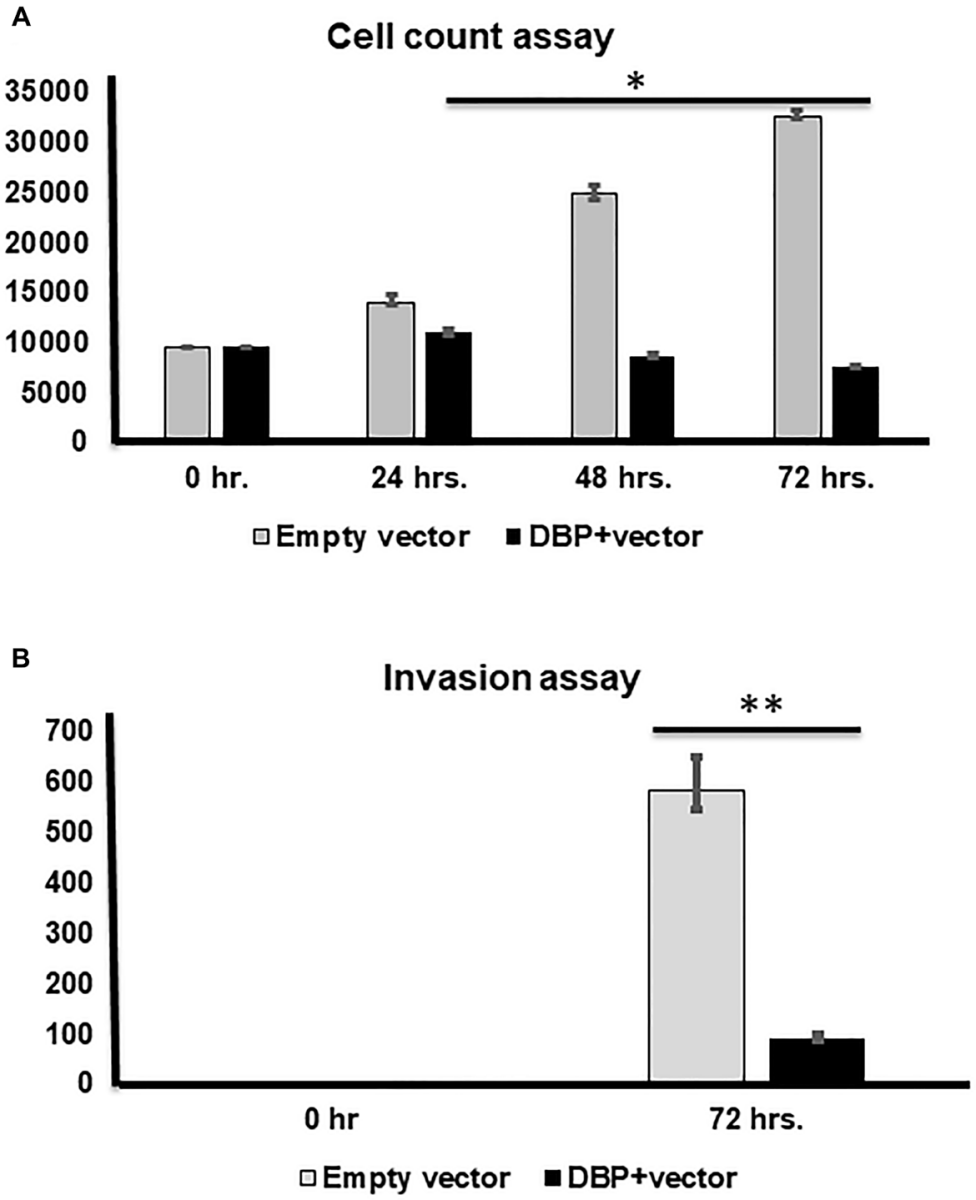
Cell counting and invasion assays after DBP-upregulation. (**A**) Cell counting at 0, 24, 48, and 72 hrs. after DBP overexpression compared to empty vector used as a control, a significantly lower cell count noted at 48 and 72 hrs. (^*^ *p* < 0.05; ^**^ *p* < 0.01; ^***^ *p* < 0.001), respectively. (**B**) A significantly lower invasion is shown after DBP-overexpression compared to empty vector at 72 hrs. (^***^ *p* < 0.001).

We achieved almost 90% knockdown/overexpression of the DBP gene in PTC cells after sorting out the positive clones and confirmed the expression level by western blotting (Supplementary Figure 3A), using actin as an internal control. After the knockdown, we found a time-dependent significantly (^*^ *p* < 0.05) higher cell rescue occurred after DBP-knockdown compared to sh-control (Figure 4A); a significantly (^**^ *p* < 0.01) higher cell migration was observed in DBP-knockdown cells compared to *sh*-control cells (Figure 4B). The reverse was noted when we overexpressed DBP-gene in the PTC cell line (Supplementary Figure 3B), a significantly (^*^ *p* < 0.05) lower cell count was noted compared to plasmid control (Figure 5A); cell migration was lowered significantly (^*^ *p* < 0.05) compared to empty vector control (Figure 5B). All data were reproduced in triplicates.

## DISCUSSION

We demonstrated a differential DBP expression in two of the most affected ethnic groups with thyroid cancer. They exhibited different amplitudes of cancer progression; notably faster progression in Filipino Americans with higher recurrence/death rates compared to European Americans. In this study, we found statistically significant (moderate to strong) DBP staining intensities in the cancer tissues from European Americans. In contrast, we observed significantly low to no DBP staining in the cancer tissues from Filipino Americans. We also determined an inverse relationship of DBP expression with cancer staging. Significantly low to no DBP staining was correlated to advance staging in Filipino American-derived cancer tissues, which showed aggressive phenotypes. Data showed a moderate to strong DBP expression that correlated to early cancer staging in most of the European Americans. These data implied that DBP's presence might play protective roles in cancer progression in European Americans compared to Filipino Americans, supporting the aggressive phenotype observed in Filipino Americans. Our data is consistent with a meta-analysis of cancers, which showed a strong correlation between higher DBP levels and better prognosis [26, 50]. DBP has been shown to act through direct/indirect pathways to attenuate TC growth [30, 31, 33–35, 37, 40, 46, 51] with higher DBP levels correlating with a better TC prognosis [30, 31, 35, 37, 39, 52]. Together, DBP plays a potential role in TC health disparities. Although we demonstrated low DBP in advanced tumors from Filipino Americans, we need to determine the progressive loss of DBP throughout TC staging.

The genomic regulation of DBP is not clearly understood. Studies show that estrogen and IL-6 increase DBP expression and enhance DBP production while TGF-β inhibits DBP production [53, 54], are also known regulators of TC oncogenesis; therefore, more in-depth studies are needed to understand their effect on DBP functionality in TC oncogenesis. Additionally, the mechanism by which the DBP gene is lost, not well understood. The DBP gene, also known as the GC gene, gives rise to alleles at different frequencies between different ethnic populations. The unique alleles are useful tools for anthropological studies that reveal ancestral links between populations [24, 55, 56]. These alleles have been associated with phenotypic differences in DBP protein structure. Although low vitamin D correlated to DBP-SNP previously [24, 57]; however, a recent study shows no association. A systemic review demonstrated that a large number of chronic diseases, including cancers, have been associated with DBP variants [29]. Therefore, we are working on to determine whether a higher frequency of DBP-variants associate to thyroid cancer in Filipino Americans versus European Americans.

Although DBP is an essential protein with multifunctional properties [28, 41–47], very few studies are available on its direct contribution to cancer cell proliferation, colony formation, and migration. This study successfully demonstrated that a stable knockdown of DBP enhanced cell proliferation and migration of PTC cells. Besides, when we overexpressed the DBP gene in the PTC cell line, we found a significant reduction in cell proliferation and migration. These data suggest a direct functional consequence of DBP-gene loss/gain-in function in thyroid cancer cell progression.

DBP is a multidomain protein. The N-terminal domain binds with vitamin D, whereas the C-terminal domain contains an O-linked glycosylation site on a threonine residue in human DBP. Selective deglycosylation of DBP occurs naturally as part of the inflammatory response. The resultant molecule, called DBP-MAF acts as a potent activator of macrophages [58, 59], which plays a role in the treatment of Ehrlich ascites tumor in mouse models [60, 61]. Administration of DBP-MAF as adjuvant immunotherapy to photodynamic therapy of cancer [37, 38], has a synergistic effect on tumor remission using a squamous cell carcinoma model in mice. It was hypothesized that DBP-MAF elicited its effect by activating macrophages, directly attacking the tumor cells. Furthermore, studies have shown that DBP-MAF elicited an antiangiogenic function. Systemic administration of DBP-MAF can inhibit the rate of tumor growth of various solid tumors and, in some cases, can cause regression of established tumors. Further characterization and study of this promising potential drug (DBP-MAF) may hasten its progress to clinical applications for patients with low DBP, including but not limited to the treatment of cancer.

In conclusion, we demonstrate that the presence or absence of DBP inversely correlates to thyroid cancer staging in two ethnicities. We report that most Filipino Americans presented with advanced thyroid cancer and showed low to no DBP expression. In contrast, European Americans with early stage PTC, showed a moderate to strong DBP expression, supporting the protective roles of DBP in the tumor microenvironment, independent of vitamin D. Our *in vitro* study details the functional consequences of loss-of/gain-in-DBP-function in thyroid cancer oncogenesis. We conclude that the gain/loss of DBP may stimulate immune-modulated signaling pathways in thyroid cancer health disparities, which awaits further investigation.

## MATERIALS AND METHODS

### Tumor samples and patient information

A total of 200 archival thyroid tissues, including 100 Filipino Americans (FA) and 100 European Americans (EA), were obtained from the Departments of Pathology at Loma Linda University Medical Center (LLUMC), VA Loma Linda Medical Center, and Riverside County Regional Medical Center (RCRMC), and Harbor UCLA Medical Center. The following are the inclusion criteria of this study: all age groups, both sex (18–75 years), collected from 2000–2019, with adequate clinical information and paraffin blocks for immunohistochemistry. All histological diagnoses were confirmed (Papillary thyroid cancer) using established morphological criteria using routine hematoxylin and eosin (H&E) staining. Patient information, including demographic data (age, BMI, and sex) (Supplementary Figure 1A and 1B), tumor size, extrathyroidal extension, nodal status, distant metastases, and disease stage (Supplementary Figure 1C and 1D), were obtained by independent chart review by our Pathologists (pathological tumor, node, metastasis; pTNM staging). All samples were obtained in IRB approved-studies according to the university and hospital policy at both Loma Linda University and Riverside County Regional Medical Centers.

### Histological examination

All histological diagnoses were confirmed using established morphological criteria using routine hematoxylin and eosin (H&E) staining as described before [1]. We included papillary thyroid cancer (PTC), the most common subtype of thyroid cancer (80–90%), to maintain genetic uniformity. Patient information, including demographic data, tumor size, extrathyroidal extension, nodal status, distant metastases, and disease stage, was obtained by independent chart review (pathological tumor, node, metastasis, pTNM staging) (Supplementary Tables 1–3).

### Analysis of DPB expression by immunohistochemistry

Formalin-fixed paraffin-embedded tissues (FPPE) were cut in 5 mm thickness. The detailed deparaffinization and immunohistochemistry protocols were described before [62, 63]. Slides were stained using the commercially available anti-DBP antibody (Novus Biologicals, CO, USA). The staining was performed as follows: the slides were deparaffinized using xylenes and graded ethyl alcohols and then rinsed in water. Next, antigen retrieval was performed by boiling slides in Antigen Retrieval Solution (Dako, Carpinteria, CA, USA; pH 6.0) in a microwave oven at maximum power for 4 min and at 50% power for 12 min, followed by a 30 min cool-down and rinsing in wash buffer. Slides were sequentially treated with the following reagents in a humidified chamber at room temperature: 10% normal rabbit serum for 30 min, anti-DBP antibody (1:100 dilution) overnight, negative control slide with PBS alone, a hepatic tissue as a positive control, and a secondary antibody conjugated with Alexa Fluor 555 for 30 min for signal amplification (wash buffer steps were included between each step). Nuclear staining was performed using DAPI containing mounting media for 5 min. Stained slides were then analyzed for DBP expression by two experts individually, and they were blinded with clinical data. Staining intensities were categorized as negative, weak, moderate, and strong (0, 1+, 2++, 3+++, respectively).

Stained tumor tissues were imaged and analyzed with an Olympus FV 1000 laser scanning confocal imaging system mounted onto an Olympus 1 × 81 microscope (Olympus America Inc., PA). Microscopic data was acquired with a 20× objective lens. Tumors were graded into categories based on staining pattern: a) no (0), b) weak (1+), c) moderate (2++), and d) strong (3+++) expression. Percent loss was calculated from a total number of cases in each ethnic group.

### Image acquisition using laser-scanning confocal microscopy

The image acquisition were followed and described previously in our published article [1]. Stained tumor tissues were imaged and analyzed with an Olympus FV 1000 laser scanning confocal imaging system mounted onto an Olympus 1 × 81 microscope (Olympus America Inc., PA, USA). Confocal images of each section were analyzed using Image-Pro (v5.9; Media Cybernetics, Silver Spring, MD). All the images were acquired in *z*-stack mode (pitch = 0.5 μm; ~15 images/stack, ~300 total images were taken from each section). Microscopic data were acquired with a 20× objective lens and two investigators (including a pathologist) working independently. Both scientists and pathologists were blinded, and subsequently, staining intensities were matched with the clinicopathological staging (Supplementary Tables 1–3).

We assessed the degree of intensity using computer-aided image classification and visual scoring by two independent pathologists. We compared pathologists annotated and software-classified areas of cancer nodules and characterized them into the following categories based on staining pattern: a) no expression: 0, b) positive expression: weak 1+, moderate 2++, and strong 3+++. Numbers and intensity of positive cancer cells were counted in each field and matched with H&E staining using image software in confocal microscopy. Fractions of the negative score (0), weakly positive (1+), moderately positive (2++), and strongly positive (score 3+++) cell estimated, fractions were multiplied with scores and summed, the total being H-score. We have counted about 300 cells sampled from 5–10 fields of vision. The positive staining rate (%) was calculated by adding all the scores in each case. Total numbers of positive cases were divided by the total number of cancer cases (same as for benign cases) and multiplied by 100. Our scoring system was combined with both intensity and distribution of positive staining. Two independent observers’ scores were entered into the database using the Lotus-1-2-3 approach software and analyzed using the weighted k statistics (kw) for interobserver error assessment.

### Assessment of staining patterns

We followed the same protocol as we described before [1]. In brief, the presence or absence of staining and depth of the color was noted by Z-stack. The number of cells showing the positive reaction and the nuclear or cytoplasmic pattern of staining was noted. Weak (1+) staining; when less than 50% of cells showed low fluorescent signal throughout the Z-stacking planes; moderate (2++) staining; when moderate signals were shown in more than 50% cells in low power and Z-stacking planes and finally, Strong (3+++) staining; when more than 50% cells showed strong signal in low power field as well as z-stacking planes).

### The effects of loss-of-/gain-in-function of DBP gene in papillary thyroid cancer cells

Papillary thyroid cancer cell lines were obtained from Dr. Frances Karr (Vermont University). Cells were used between passages 5 and 10. After resuscitation, cell lines were routinely authenticated (once every 6 months), through cell morphology monitoring, growth curve analysis, species verification by isoenzymology and karyotyping, identity verification using short tandem repeat profiling analysis, and contamination checks., as described before [64]. Cells were grown at 37°C and 5% CO2 in Dulbecco’s modified Eagle’s medium (DMEM), RPMI or DMEM/Ham’s F12 1:1 (Gibco) with 10% fetal bovine serum (FBS). DBP cDNA (RG202051), knockdown plasmids expressing short hairpin RNA (shRNA) targeting DBP (sc-41375-SH), and control shRNA plasmids (Santa Cruz Biotech) were purchased. Control (shControl) or DBP knockdown (shDBP) plasmids were transfected into the PTC cell line using shRNA plasmid transfection reagent (Santa Cruz Biotech) according to the instruction manual to produce stable clones. The stable transfectants were selected in 500 μg/mL puromycin (Sigma-Aldrich) after 24 hours. At every 3 days interval the selection medium was replaced, for a period of 2 weeks. Subsequently, clones of resistant cells were isolated and allowed to proliferate in a medium containing puromycin (500 μg/mL).

### Determine DBP-transfection efficiency by western blot analysis

Western blot was performed according to the method described before [65]. Cells were washed in PBS and then lysed in a lysis buffer with 50 mmol/L Tris-HCl (pH 7.5), 150 mmol/L NaCl, 1 mmol/L EDTA, 1 mmol/L MgCl2, and 0.5% Triton X-100. The lysates were cleared by centrifugation at 13,000 × *g* for 20 minutes at 4°C. The lysates were separated by SDS-PAGE, transferred to polyvinylidene difluoride membranes, and probed with the DBP (ThermoFisher Scientific) and actin (Cell Signaling) antibodies. The signals were detected by Li-Cor.

### Determine cell proliferation after DBP-overexpression

The cell culture was performed in a humidified incubator (95% air, 5% CO2, 37°C) in 96-well flat-bottomed microtiter plates for 24, 48, 72, and 96 hours. At each time point, the number of viable cells was counted using the 3-(4, 5-dimethylthiazol-2-yl)-2,5-diphenyltetrazolium bromide (MTT; Sigma-Aldrich) assay by monitoring the absorbance at 570 nm. Cell doubling time was calculated using the formula in the technical information for working with animal cells in culture, provided by ATCC.

### Cell migration and invasion assay after DBP-transfection

Collagen cell migration assay was performed on transfected cells using the QCM 96-well fluorometric collagen-based cell invasion assay (Millipore) according to the manufacturer’s instructions to measure TC invasion as described before [65].

### Statistical analysis

DBP expression levels were determined by contingency table analysis and Chi-square test. Age- and sex-adjusted prevalence ratios were obtained with exact Poisson regression. Data were analyzed using SPSS (version 17.0; SPSS Inc.). Interval variables are presented as the mean +/– SEM or median +/– interquartile range. The comparisons for statistical significance were performed with level of significance set at *p* < 0.05. STATA v14 (StataCorp LLC; College Station, TX, USA) was used for all analyses.

## SUPPLEMENTARY MATERIALS


