# Supplementary material for: Differential expression of Vitamin D binding protein in thyroid cancer health disparities
Source: Oncotarget. 2021 Mar 30;12(7):596–607. doi: 10.18632/oncotarget.27920 (PMC8021030; doi:10.18632/oncotarget.27920)
Supplement: Supplementary file 1 [file oncotarget-12-596-s001.pdf]

Differential expression of Vitamin D binding protein in thyroid cancer health disparities

SUPPLEMENTARY MATERIALS

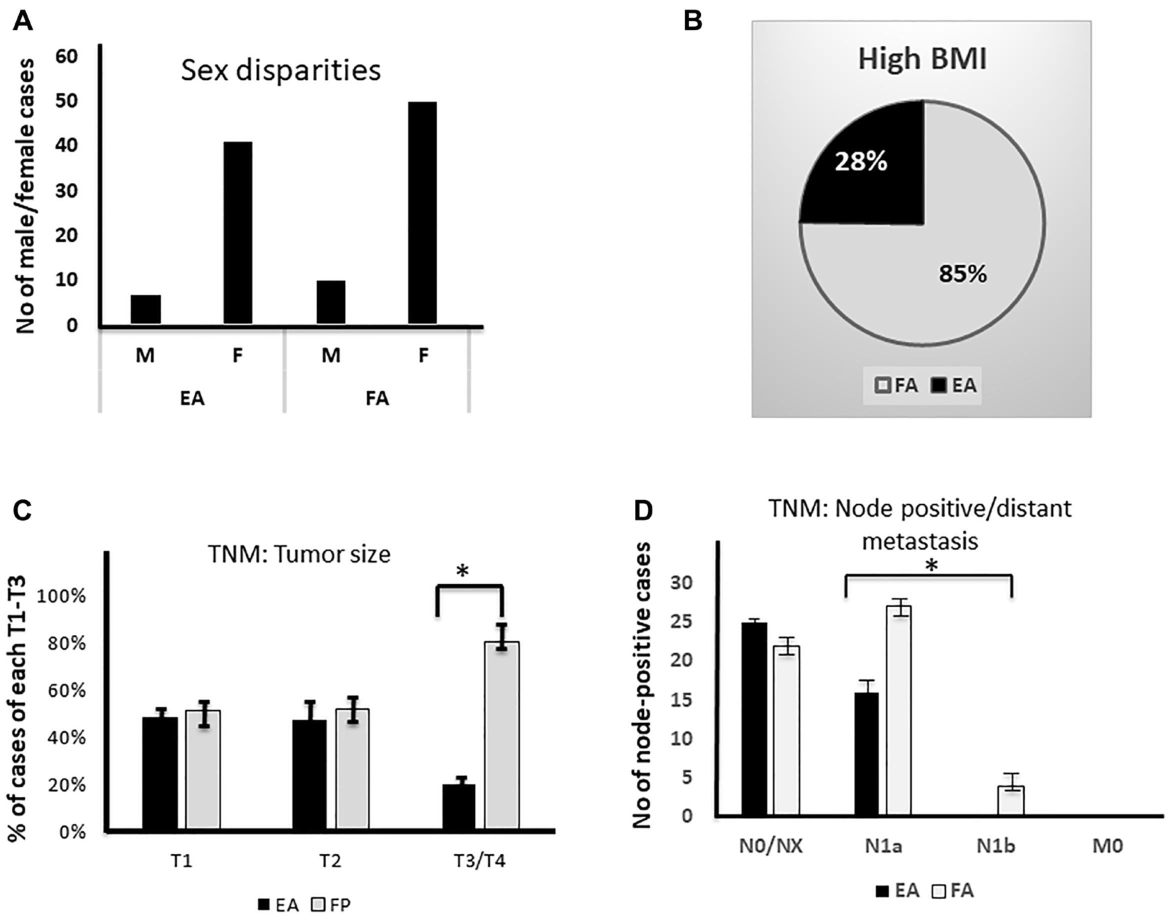

Supplementary Figure 1: Disparities in sex, BMI, and TNM staging are observed. (**A**) A higher ratio of female to male is observed in both ethnicities; (**B**) A higher rate of BMI (high) was observed in FA (85%) compared to EA (28%); (**C**) A higher rate of tumor size (T3/T4) is observed in FA compared to EA ($^*p < 0.05$); (**D**) a significantly higher node positive cases were seen in FA compared to EA, however no distant metastasis (M0) were found in these cases.

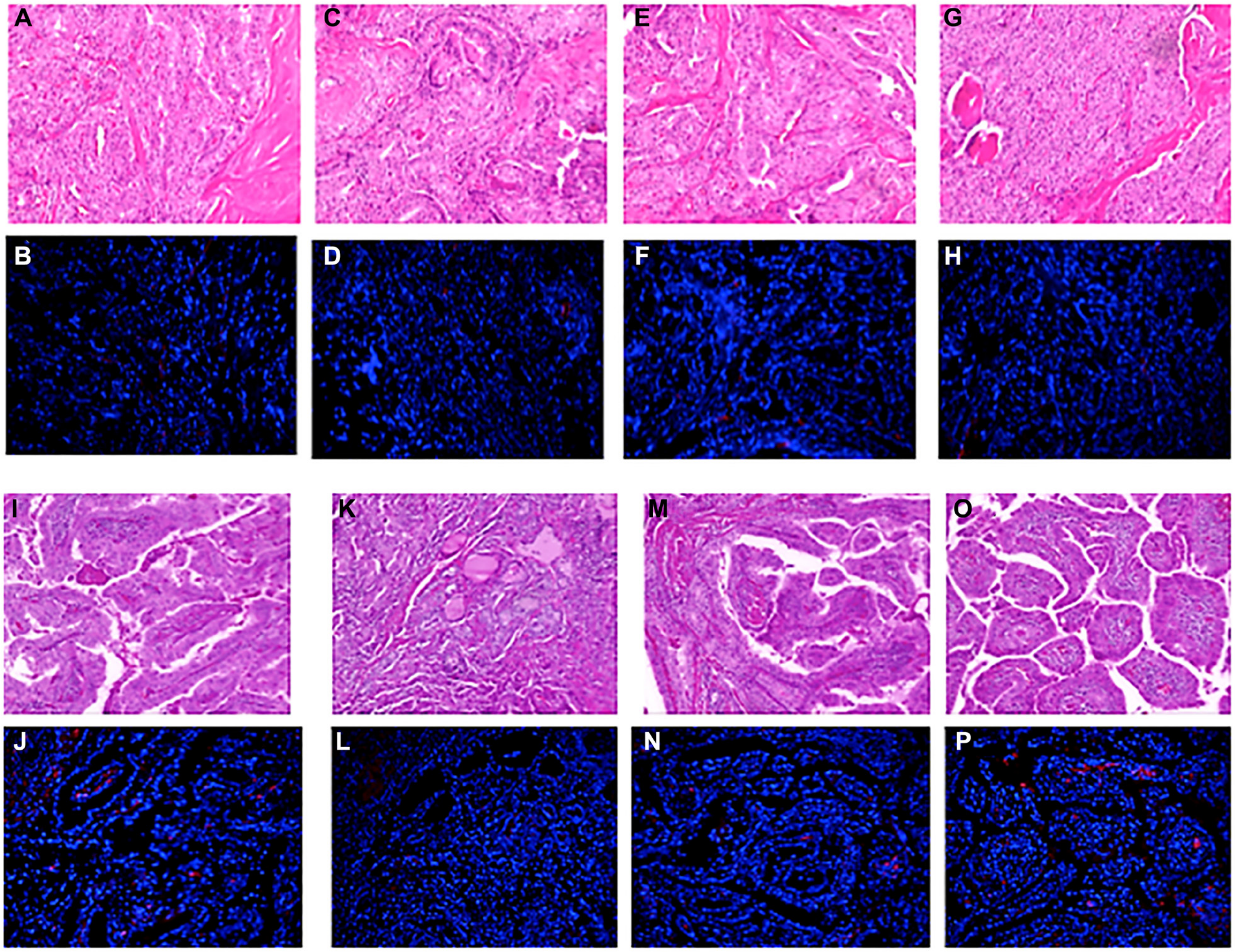

Supplementary Figure 2: Hematoxylin and eosin (H&E) staining and immunohistochemistry of DBP in FPTC. (**A**–**D**) Shows H&E staining of FPTC (1–4 cases); (**E**–**H**) shows confocal microscopy of DBP staining of the corresponding cases (1–4 cases); (**I**–**L**) Shows H&E staining of FPTC (5–8 cases); (**M**–**P**) shows confocal microscopy of DBP staining of the corresponding cases (5–8 cases). (Original magnification 10×). FATC, Filipino American-derived thyroid cancer; DBP, vitamin D binding protein.

A

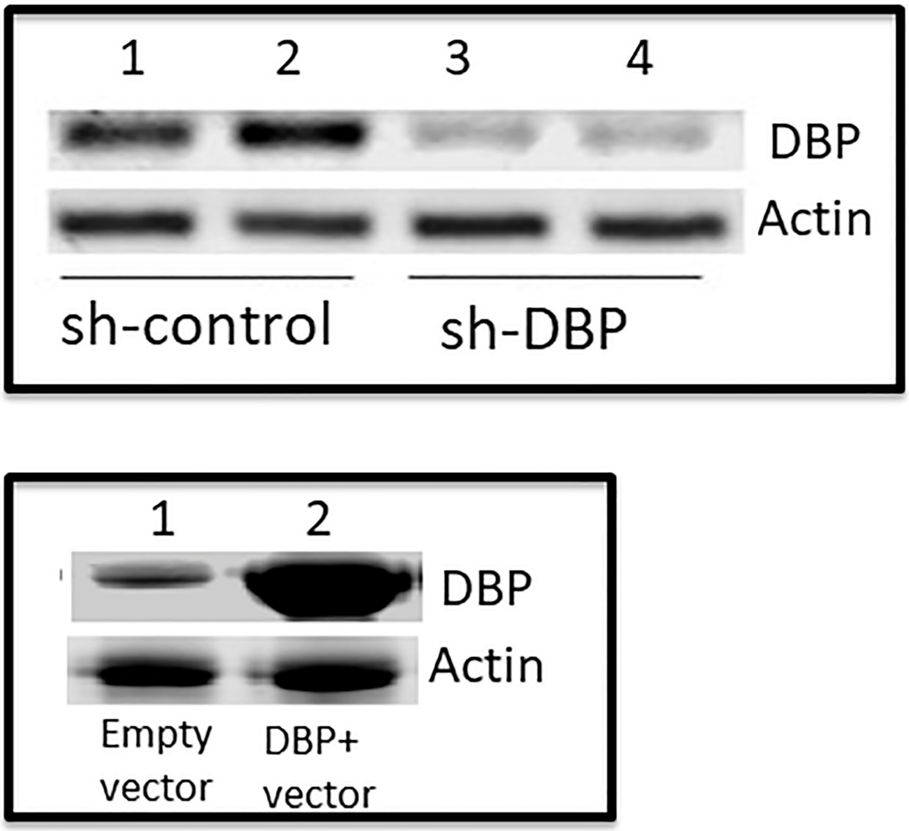

B

Supplementary Figure 3: Efficiency of knock-down and overexpression of DBP gene in human PTC cell lines. Western blot analysis was done after 72 hours of transfection; (**A**) PTC cells is transfected with si-scramble (1, 2) and si-DBP (3, 4), western blot was performed with anti-DBP (top panel) and anti-actin antibodies (bottom panel). (**B**) PTC cells were transfected with empty vector (1) and DBP-transduced vector (2), western blot was performed with anti-DBP (top panel) and anti-actin antibodies (bottom panel) after 72 hrs. of transfection.

Supplementary Table 1: Patient demographic of TNM (T1) staging with age, sex, BMI with DBP protein expression Filipinos vs. Europeans

Patient no	Age	Sex	Ethnicity	Type of thyroid cancer	pTNM	High BMI	DBP staining
Pt#3	28	M	Asian (FA)	PTC	pT1aN1a (Stage III)	+	0
Pt#4	45	F	Asian (FA)	PTC (microcarcinoma)	pT1aNX (Stage I)	+	+
Pt#7	55	F	Asian (FA)	Follicular variant of PTC	pT1aNX (Stage I)	–	+
Pt#8	24	F	Asian (FA)	Follicular variant of PTC	pT1aNX (Stage I)	+	+
Pt#9	55	M	Asian (FA)	Follicular variant of PTC	pT1aNX (Stage I)	+	+
Pt#13	36	F	Asian (FA)	PTC	pT1bN0 (Stage I)	+	+
Pt#16	48	F	Asian (FA)	PTC	pT1aN1a (Stage II)	+	0
Pt#18	49	F	Asian (FA)	PTC	pT1bN1b (Stage III)	+	0
Pt#20	37	F	Asian (FA)	PTC	pT1aNX (Stage II)	+	0
Pt#22	35	M	Asian (FA)	PTC	pT1aN0 (Stage I)	+	+
Pt#24	74	M	Asian (FA)	Follicular variant of PTC	pT1aNx (Stage I)	–	0
Pt#27	50	F	Asian (FA)	PTC	pT1a NX (Stage I)	+	0
Pt#30	33	F	White	PTC	pT1a N0 (Stage I)	–	+++
Pt#33	34	F	White	PTC	pT1a NX (Stage I)	–	+++
Pt#35	43	F	White	PTC	pT1aNX (Stage I)	–	++
Pt#38	80	M	White	PTC	pT1a pN1a (Stage III)	–	++
Pt#39	45	F	Asian (FA)	PTC	pT1b pN1a (Stage I)	+	+
Pt#43	74	F	White	Follicular variant of PTC	pT1aNX (Stage II)	+	++
Pt#48	69	F	White	Micropapillary carcinoma	pT1NX (Stage I)	–	+++
Pt#49	33	F	White	PTC	pT1a N0 (Stage I)	+	+
Pt#50	49	F	White	PTC	pT1a N0 (Stage I)	–	+
Pt#52	34	F	White	PTC	pT1a N1 (Stage I)	–	+++
Pt#57	43	F	Asian (FA)	PTC	pT1aNX (Stage I)	+	0
Pt#60	80	M	White	PTC with microcalcification	pT1a pN1a (Stage III)	–	+
Pt#61	45	F	Asian	PTC	pT1b pN1a (Stage II)	–	0
Pt#37	74	F	White	PTC with calcification	pT1 NX (Stage I)	–	+
Pt#28	75	M	White	PTC with microcalcification	pT1a(m) N1a MX (Stage III)	–	++
Pt#15	45	M	Asian (FA)	Follicular variant of PTC	pT1aNX (Stage I)	+	+
Pt#12	35	F	Asian (FA)	PTC with microcalcification	pT1N1a (Stage II)	+	0
Pt#10	23	F	Asian (FA)	PTC with microcalcification	pT1bN1a (Stage I)	+	0
Pt#46	50	F	Asian (FA)	PTC	pT1a NX (Stage II)	+	0
Pt#48	59	F	White	Micropapillary carcinoma	pT1NX (Stage I)	–	+++
Pt#49	33	F	White	PTC	pT1a N0 (Stage I)	–	+++
Pt#50	49	F	White	PTC	pT1a N0 (Stage I)	–	+
Pt#52	34	F	White	PTC	pT1a N1 (Stage II)	+	++
Pt#57	43	F	Asian (FA)	PTC	pT1aNX (Stage I)	+	+
Pt#61	45	F	Asian (FA)	PTC	pT1b pN1a (Stage II)	–	0
Pt#63	35	F	Asian (FA)	PTC	pT1aN0 (Stage I)	–	0
Pt#65	74	F	White	Follicular variant of PTC	pT1aNX (Stage I)	+	+++
Pt#68	50	F	Asian (FA)	PTC	pT1a NX (Stage I)	+	0

Pt#69	75	M	White	PTC with microcalcification	pT1a(m) N1a MX (Stage II)	+	0
Pt#71	33	F	White	PTC	pT1a N0 (Stage I)	–	+++
Pt#72	49	F	White	PTC	pT1a NX (Stage I)	–	+++
Pt#73	27	F	Asian (FA)	PTC with microcalcification	pT1 NX (Stage I)	+	+
Pt#74	34	F	White	PTC	pT1a NX (Stage I)	+	+++
Pt#74	43	F	White	PTC	pT1NX (Stage I)	+	++
Pt#78	45	F	Asian (FA)	PTC	pT1b pN1a (Stage II)	+	+
Pt#82	25	F	Asian (FA)	PTC	pT1aN0 (Stage I)	+	0
Pt#84	74	F	White	Follicular variant of PTC with calcification	pT1aNX (Stage I)	–	+++
Pt#86	24	F	White	Follicular carcinoma	pT1N0 (Stage I)	–	+++
Pt#87	50	F	Asian (FA)	PTC	pT1a NX (Stage I)	+	0
Pt#88	75	M	White	PTC with calcification	pT1a(m) N1a Mx (Stage III)	+	++

Pt#, patient number; F, female; M, male; p, pathological; T, tumor size (T1a Tumor 1 cm or less, limited to the thyroid; T1b: Tumor more than 1 cm but not more than 2 cm in greatest dimension, limited to the thyroid; T2; Tumor more than 2 cm but not more than 4 cm in greatest dimension, limited to the thyroid; T3: Tumor more than 4 cm in greatest dimension, limited to the thyroid or any tumor with minimal extrathyroid extension (e.g., extension to sternothyroid muscle or perithyroid soft tissues); N, Regional node; NX, Regional lymph nodes cannot be assessed; N0, No regional lymph node metastasis; N1, Regional lymph node metastasis; N1a, Metastasis to Level VI (pretracheal, paratracheal, and prelaryngeal lymph nodes); N1b, Metastasis to unilateral, bilateral, or contralateral cervical Levels I, II, III, IV, or V) or superior mediastinal lymph nodes (Level VII) M, metastasis; M0, No distant metastasis; M1, Distant metastasis. +, yes; –, no. VDBP, vitamin D binding protein; PTC, papillary thyroid cancer; FA, Filipinos; M, male; F, female.

Supplementary Table 2: Patient demographic of TNM (T2) staging with age, sex, BMI with DBP protein expression Filipinos vs. Europeans

Patient No	Age	Sex	Ethnicity	Type of thyroid cancer	pTNM	High BMI	DBP staining
Pt#2	25	F	Asian (FA)	PTC	pT2N1a (Stage II)	+	0
Pt#11	36	F	Asian (FA)	PTC with microcalcification	pT2N1a (Stage II)	–	0
Pt#17	49	F	Asian (FA)	PTC with microcalcification	pT2NX (Stage II)	+	0
Pt#19	79	F	Asian (FA)	Follicular variant of papillary carcinoma microcalcification	pT2N0 (Stage III)	+	0
Pt#23	20	F	Asian (FA)	PTC with calcification	pT2NX (Stage II)	+	0
Pt#29	59	M	White	Micropapillary carcinoma with calcification	pT2N1a (Stage III)	+	++
Pt#31	49	F	White	PTC with microcalcification	pT2a N1 (Stage III)	+	++
Pt#32	77	F	Asian (FA)	PTC	pT2 Nx (Stage II)	–	0
Pt#34	26	F	White	PTC with microcalcifcation	pT2NX (Stage I)	+	+++
Pt#41	65	M	Asian (FA)	PTC	pT2N0 (Stage II)	+	0
Pt#42	20	F	Asian (FA)	PTC	pT2NX (Stage I)	–	0
Pt#51	77	F	Asian (FA)	PTC with microcalcification	pT2 NX (Stage II)	+	+
Pt#59	74	F	White	PTC	pT2 NX (Stage II)	–	++
Pt#64	20	F	Asian (FA)	PTC with microcalcification	pT2NX (Stage I)	+	+
Pt#70	59	F	White	Micropapillary carcinoma with microcalcification	pT2N1 (Stage III)	+	++
Pt#75	26	F	White	PTC	pT2NX (Stage I)	–	+++
Pt#76	74	F	White	PTC	pT2 NX (Stage II)	–	++
Pt#83	20	F	Asian (FA)	PTC with microcalcification	pT2NX (Stage I)	+	+
Pt#92	77	F	Asian (FA)	PTC	pT2 NX (Stage II)	+	0
Pt#93	34	F	White	PTC	pT2a N1 (Stage I)	–	+++
Pt#94	26	F	White	PTC	pT2NX (Stage I)	+	++
Pt#95	43	F	White	PTC	pT2NX (Stage I)	+	++
Pt#97	74	F	White	PTC	pT2 NX (Stage II)	–	++

Pt#, patient number; F, female; M, male; p, pathological; T, tumor size (T1a Tumor 1 cm or less, limited to the thyroid; T1b: Tumor more than 1 cm but not more than 2 cm in greatest dimension, limited to the thyroid; T2; Tumor more than 2 cm but not more than 4 cm in greatest dimension, limited to the thyroid; T3: Tumor more than 4 cm in greatest dimension, limited to the thyroid or any tumor with minimal extrathyroid extension (e.g., extension to sternothyroid muscle or perithyroid soft tissues); N, Regional node; NX, regional lymph nodes cannot be assessed; N0, no regional lymph node metastasis; N1, regional lymph node metastasis; N1a, Metastasis to Level VI (pretracheal, paratracheal, and prelaryngeal lymph nodes); N1b, Metastasis to unilateral, bilateral, or contralateral cervical Levels I, II, III, IV, or V) or superior mediastinal lymph nodes (Level VII) M, metastasis; M0, No distant metastasis; M1, Distant metastasis. +, yes; –, no. DBP, vitamin D binding protein; PTC, papillary thyroid cancer; FA, Filipinos; M, male; F, female.

Supplementary Table 3: Correlation of clinicopathological features with TNM (T3) staging age, sex, with DBP protein expression in Filipinos vs. Europeans

Patient No	Age	Sex	Ethnicity	Types of thyroid cancer	pTNM	High BMI	DBP Staining
Pt#1	72	F	Asian (FA)	PTC with Follicular variant	pT3N1b (stage IV)	+	0
Pt#5	70	M	Asian (FA)	PTC with microcalcification	pT4aN1b (Stage IV)	−	0
Pt#6	40	F	Asian (FA)	PTC/ATC microcalcification	pT4aN1b (Stage IV)	+	0
Pt#14	25	F	Asian (FA)	PTC with Follicular variant with calcification	pT3N1a (Stage I)	+	0
Pt#21	66	M	Asian (FA)	PTC with Follicular variant with calcification	pT3N1a (Stage III)	−	0
Pt#25	34	F	Asian (FA)	PTC with Follicular variant with calcification	pT3 N1 (Stage III)	+	0
Pt#26	24	F	Asian (FA)	PTC with Follicular variant with calcification	pT3N1a (Stage III)	+	0
Pt#36	89	M	White	PTC with microcalcification	pT3 (m) pN1a (Stage III)	−	++
Pt#40	46	M	Asian (FA)	PTC with calcification	pT3N1a (Stage II)	+	0
Pt#44	84	F	White	PTC with Follicular variant	pT3 N1 (Stage III)	−	++
Pt#45	74	F	White	PTC with Follicular variant	pT3N1a (Stage III)	−	++
Pt#53	66	F	White	PTC	pT3N1 (Stage III)	+	+++
Pt#54	32	F	Asian (FA)	PTC	pT3N1a (Stage III)	+	0
Pt#55	84	F	Asian (FA)	PTC with Follicular variant	pT3N1a (Stage III)	−	0
Pt#56	47	F	Asian (FA)	PTC with microcalcification	pT3N1aM0 (Stage III)	+	0
Pt#58	42	F	Asian (FA)	PTC with calcification	pT3 (m) pN1a (Stage III)	+	0
Pt#62	76	M	Asian (FA)	PTC with microcalcification	pT3N1a (Stage III)	−	0
Pt#66	74	F	White	PTC with microcalcification	pT3 N1 (Stage III)	−	++
Pt#67	24	F	Asian (FA)	PTC microcalcification	pT3N1a (Stage III)	+	0
Pt#75	42	F	Asian (FA)	PTC with microcalcification	pT3 (m) pN1a (Stage III)	+	0
Pt#79	40	F	Asian (FA)	PTC with calcification	pT3N1a (Stage I)	−	0
Pt#80	51	F	Asian (FA)	PTC with microcalcification	pT3N1a (Stage III)	+	0
Pt#81	86	M	Asian (FA)	PTC with microcalcification	pT3N1 (Stage III)	−	0
Pt#85	24	F	Asian (FA)	PTC with Follicular variant with calcification	pT3 N1 (Stage I)	+	0
Pt#96	42	F	Asian (FA)	PTC with microcalcification	pT3 (m) N1a (Stage II)	+	0

Pt#, patient number; F, female; M, male; p, pathological; T, tumor size (T1a Tumor 1 cm or less, limited to the thyroid; T1b: Tumor more than 1 cm but not more than 2 cm in greatest dimension, limited to the thyroid; T2; Tumor more than 2 cm but not more than 4 cm in greatest dimension, limited to the thyroid; T3: Tumor more than 4 cm in greatest dimension, limited to the thyroid or any tumor with minimal extrathyroid extension (e.g., extension to sternothyroid muscle or perithyroid soft tissues); T4 All anaplastic carcinoma are considered T4 tumors; N, Regional node; NX, Regional lymph nodes cannot be assessed; N0, No regional lymph node metastasis; N1, Regional lymph node metastasis; N1a, Metastasis to Level VI (pretracheal, paratracheal, and prelaryngeal lymph nodes); N1b, Metastasis to unilateral, bilateral, or contralateral cervical Levels I, II, III, IV, or V) or superior mediastinal lymph nodes (Level VII) M, metastasis; M0, No distant metastasis; M1, Distant metastasis. +, yes; –, no. VDBP, vitamin D binding protein; SNP, single nucleotide polymorphism.
